# Wastewater remediation of pharmaceuticals with ozone and granular active carbon: a risk-driven approach

**DOI:** 10.1039/d5ew00600g

**Published:** 2025-10-03

**Authors:** Charlie J. E. Davey, Asmita Dubey, Pia Keutmann, Thomas L. ter Laak, Lisette de Senerpont Domis, Annemarie P. van Wezel

**Affiliations:** a Institute for Biodiversity and Ecosystem Dynamics, University of Amsterdam Amsterdam the Netherlands c.j.e.davey@uva.nl; b Department of Aquatic Ecology, Netherlands Institute of Ecology (NIOO-KNAW) Droevendaalsesteeg 10 6708 PB Wageningen the Netherlands; c Institute for Analytical Research, Hochschulen Fresenius Gem. Trägergesellschaft MbH Idstein Germany; d KWR Water Research Institute Nieuwegein the Netherlands; e Faculty of EEMCS and ITC, University of Twente the Netherlands; f Faculty of Geosciences, Utrecht University the Netherlands

## Abstract

This study aimed to investigate the removal efficiency of (psycho)pharmaceuticals by ozonation and granular active carbon (GAC) in wastewater effluent, using risk as the metric for adequate removal instead of aqueous concentrations. Conventionally treated effluent was further treated with ozone or GAC until there was a 25% reduced UVA_254_ absorbance, to allow for a direct comparison of the two treatment types. Samples were analysed using Ultra High-Performance Liquid Chromatography-Quadrupole Time of Flight-High Resolution Mass Spectrometry (UHPLC-qTOF-HRMS), where 20 (psycho)pharmaceuticals were quantified, and their risk was assessed using Predicted No Effect Concentrations (PNECs). A further assessment was performed using Quantitative Structural Activity Relationships (QSARs) for both parent compounds and their Oxidation Transformation Products (OTPs) to compare the relative toxicity of new species being formed by the ozone treatment. The total median removal efficiency across all compounds was 60 ± 3% for ozone in terms of concentration, yielding a 73 ± 2% reduction in terms of risk for the parent compounds, while the median removal efficiency for GAC is 57 ± 9% as expressed in concentration, and 46 ± 11% in terms of risk reduction. When factoring in the OTP toxicity, the median risk reduction for ozone flips to −274 ± 124%, indicating that there may be an increase in risk during ozonation. Pearson correlations on molecular descriptors indicated that ozone removal most strongly correlated with the number of activated aromatic rings (*r* = 0.65), while for GAC the topological polar surface area correlated strongest with removal (*r* = 0.54), therefore indicating that ozone and GAC target different types of molecules. The study demonstrates the merits of a risk-driven approach over concentration-based removal targets in current legislation, but also highlighted some drawbacks, especially with regards to data gaps and model accuracies.

## Abstract 2

Ozone removed (psycho)pharmaceuticals slightly better than GAC. However, a risk-driven approach indicated that the expected creation of oxidative transformation products the modelled risk to the environment may actually increase after ozonation.

## Graphical abstract

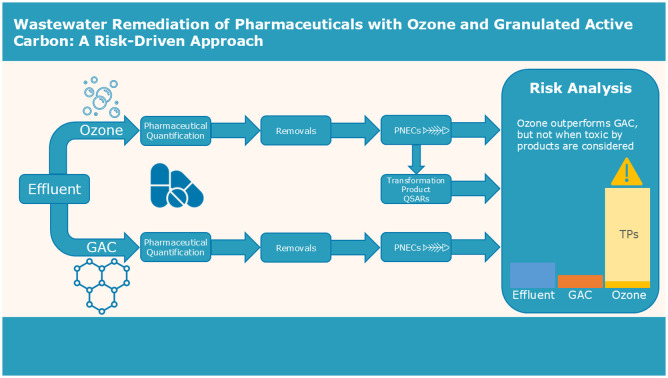

**Water impact**

Using a risk-driven approach, it was found that ozone removed (psycho)pharmaceutical parent compounds from wastewater effluent slightly better than granulated active carbon. However, due to the expected creation of oxidative transformation products the risk to the environment may actually increase after ozonation.

## 1. Introduction

Pharmaceuticals have become essential in modern medicine, contributing to increased physical and mental health, and to a higher standard of living for many people around the world.^1^ Psychopharmaceuticals, defined by the World Health Organisation under the Anatomical Therapeutic Class of N (ATC-N),^2^ are a subclass of pharmaceuticals designed to treat medical conditions related to the nervous system, often by altering the neurochemistry of the human brain.^3,4^ A growing number of people use psychopharmaceuticals on a regular basis, fuelled by several factors such as growing and aging populations, a loss of social stigmas of the underlying diseases (*e.g.* depression), and a growing number of psychopharmaceuticals entering global markets.^5–10^

In surface waters, psychopharmaceuticals have been shown to be a unique class of contaminants by exerting both lethal and sub-lethal adverse effects on many aquatic species, which is due to the similar neurochemical architecture shared between humans and other non-target species.^11,12^ Importantly, the sub-lethal effects of psychopharmaceuticals can alter the behaviour of non-target organisms, leading to changes in feeding, social interactions, predator avoidance, and locomotion, amongst other effects.^13–19^ These ecotoxicological effects have been demonstrated at environmentally relevant concentrations.^18^ Although an absence of effects has also been demonstrated.^20^ Despite large data gaps still hindering proper risk assessment, it is evident that the presence of psychopharmaceuticals can pose risks to the aquatic ecosystem.^16,21–24^

Psychopharmaceuticals enter the aquatic environment through wastewater discharges due to insufficient removal by Wastewater Treatment Plants (WWTPs).^25–27^ Since psychopharmaceuticals are often designed for chronic use due to the types of conditions they treat (*e.g.* depression), they tend to have long metabolic half-lives, ranging from days (*e.g.* carbamazepine) to weeks (*e.g.* fluoxetine) opposed to common pharmaceuticals with much shorter half-lives in the order of minutes to hours,^28^ which in turn leads to lower biodegradation rates in traditional WWTPs.^29^ Conventional Activated Sludge (CAS) WWTPs usually consist of a combination of a primary sedimentation step, aeration/activated sludge step, followed by a secondary sedimentation step. These WWTPs are mainly designed to remove nutrients and reduce biochemical oxygen demand and are reliant on adsorption to sludge and biodegradation to remove the bulk of micropollutants, which is why many psychopharmaceuticals still enter the aquatic environment.^25,30–32^ Removal efficiencies of many (psycho)pharmaceuticals by conventional treatment methods do not meet targets,^25^ and some psychopharmaceuticals can demonstrate concentration-based removal efficiencies of less than 50% in modern WWTPs.^25,32^ While WWTP effluent is not the only contributing factor to occurrence of (psycho)pharmaceuticals in the environment, with intermittent sources including storm overflow, spillage, and dumping,^33^ it remains the primary and continuous source of (psycho)pharmaceuticals into the environment.^33–38^

Advanced treatments in WWTPs include at least one additional step after conventional CAS treatment,^39–41^ and have demonstrated significantly higher removals for many micropollutants when compared to conventional treatments,^42^ albeit with increases to the capital, operation, and maintenance costs of WWTPs.^40,43^ Advanced treatment is a broad term that can refer to multiple different treatment techniques, such Advanced Oxidation Processes (AOPs), which may use ozone or ultra-violet light (UV) in combination with H_2_O_2_ and other catalysts.^44–46^ Another example of advanced treatments are adsorption/biodegradation-based processes, such as sand filtration and active carbon (granular or powdered).^47–51^ In addition, so-called nature-based solutions (NBS) may also be considered advanced treatments, which refer to techniques that utilise natural systems and biota, such as natural and constructed wetlands or algae, to bind and biodegrade the micropollutants, and try to reduce the capital and operational costs.^52–57^

Unlike the adsorptive techniques, AOPs are known to produce by-products, known as Oxidative Transformation Products (OTPs), which can be toxic to aquatic ecosystems, and their potential formation may negate the environmental benefits from parent compound removal.^58–60^ In response, AOPs are generally only used if an additional biofilter is used afterwards, such as GAC or sand filtration. While analytical advances do allow for the semi-quantification of transformation products, including OTPs,^59^ there is still a lack of data for OTPs, especially for individual OTPs.^60,61^ Without accurate occurrence data or ecotoxicity data, risk assessment on individual OTPs remains a challenge, with most OTP toxicity studies focusing on mixture bioassays.^60–66^

However, while inaccurate and only used by some environmental risk assessors as a last resort,^67^ and not by others,^68^ Quantitative Structural Activity Relationships (QSAR) models can be used to provide an estimation of the ecotoxicity of a compound provided the structure of the compound is known, and therefore provide a qualitative, preliminary risk assessment. This type of risk assessment would not be comparable to assessments used to derive environmental quality standards,^68–70^ but would advance the scientific discussion on OTPs, specifically through the lens of prioritisation. This approach would also compliment other risk-based approaches to psychopharmaceutical remediation.^25^

In recent years, there has been a growing consensus for the need for advanced treatments to help to further remove micropollutants, including (psycho)pharmaceuticals, from wastewater effluent.^27,49,71,72^ The EU's revised urban wastewater treatment directive^73^ progressively more rigorous measures over time, starting from 2025 for WWTPs that are connected to populations of over 150 k to include advanced treatment technologies. The UWWTD also sets concentration-based removal targets of 80% for 12 guide substances once quaternary (advanced) treatment has been implemented. The UWWTD leaves room for a ‘risk-based approach’ to determine if advanced treatment is needed for smaller WWTPs in certain circumstances, such as an outlet into a sensitive ecosystem affecting sources for drinking water or into a system with low dilution. Discussions are ongoing outside the UWWTD regarding the removal targets, with risk-based removal targets seen as a viable option provided that enough high quality ecotoxicity data are available^23,73^ especially when compared to concentration-based removal targets.^44,49,71,74^

While studies have shown that both adsorptive and AOP methods can yield high removals for psychopharmaceuticals^44,46,49,75–77^ there have been concerns about the lack of comparability between different advanced treatment techniques due to inconsistent experimental approaches.^45,78^ Notably, removal efficiencies between adsorptive and oxidative methods may be quite different based on the specific physicochemical properties of the compounds,^76,79^ yet determining which advanced treatment is better placed to remove these compounds become complex due to differing dosages used in those experiments.^45–48,50,65,70,72,76,77,79–82^ One method to harmonise the results to allow direct comparison of removals by different technologies is the UVA_254_ method developed by Altmann *et al.*,^47^ which has been validated in recent lab- and pilot-scale ozone experiments^82,83^ and active carbon experiments.^48^ Here, the UV absorbance at 254 nm is measured during treatment until there is predetermined drop in absorbance (*e.g.* 25%) correlating to a drop in micropollutant concentration. Direct comparison of advanced treatments is needed for better decision making around scaling to pilot setups, which has been hindered by the lack of homogeneity of experimental setups.^45,78^

Given that psychopharmaceuticals have unique modes of action, their use is increasing, and current WWTP removals are sub-optimal, further investigation into advanced treatment techniques to reduce the impact of these compounds on the aquatic environment is imperative. As such, risk assessments can be used to evaluate environmental impact and can aid in the understanding of the impact of OTPs. Therefore, the present study aimed to test the risk-based removal efficiencies for psychopharmaceuticals for ozone and GAC as two commonly used advanced treatment methods. To this end, firstly a preliminary risk assessment as an indicator for successful remediation of psychopharmaceuticals was employed, and secondly a further risk analysis was performed on OTPs from ozonation using a simple mass balance approach coupled with ecotoxicity QSAR models.

## 2. Methods

### 2.1. Materials and target list

The target list included 30 psychopharmaceuticals, following the method developed by Davey *et al.*,^25^ which are all from the ATC-N class of pharmaceuticals. We complemented this list by adding 5 pharmaceuticals that are not considered psychopharmaceuticals (ATC-N), but are highly studied, to maximise comparability to literature data (SI-1, column K). The isotopically labelled and unlabelled internal standards (SI-1) used for both the method optimization and measurements were purchased from Sigma-Aldrich Chemie (Schnelldorf, Germany). Stock solutions were prepared using MeOH and stored at −20 °C. Oasis HLB cartridges (6 cc, 150 mg) were purchased from Waters (Etten-Leur, the Netherlands). The solvents used for solid phase extraction (SPE), chromatographic separation and stock solutions were of LC-MS grade from Biosolve (Valkenswaard, the Netherlands), and ultra-pure water used for SPE, and separation was produced by a Milli-Q® Direct Water Purification System from Merck (Damstadt, Germany).

### 2.2. Samples & Treatments

Ozone and GAC were chosen as advanced treatment types, since these are commonly applied.^27,39–41^ Effluent samples were obtained from the Bennekom wastewater treatment plant (51.997205, 5.658341) on 02/08/2022 and 03/08/2022. The treatment plant uses a CAS setup and is connected to a population of 20 000 inhabitant equivalents^80^ and has been used for other effluent polishing experiments.^54^ The ozone and GAC treatment of the effluent was carried out at the Netherlands Institute of Ecology (NIOO-KNAW, Wageningen, the Netherlands) on the same day as sampling. Two effluent batches of 1200 L and 1350 L were collected for further treatment with ozone and GAC respectively (sample names PreOZ and PreGAC). These batches were subsequently used in another study for long-term mesocosm experiments to study non-lethal food web effects of differently treated effluents, hence the large volumes.

The effluent batches were treated until there was a 25% reduction of UV absorbance at 254 nm (UVA_254_), which allowed a straightforward method for comparing removal efficiency of trace contaminants across different advanced treatment methods.^47,84^ A 25% reduction in UVA_254_ corresponds with an ozone dosage of approximately 0.2–0.5 g O_3_ g^−1^ DOC and a GAC dosage of approximately 4 g L^−1^ and indicates average organic micropollutant removals of >80% based on previous experiments.^47,82,83^ The dissolved organic carbon (DOC) concentration of the wastewater before treatment was 10 mg L^−1^.

Four effluent batches of 300 L were ozonated with an ozone generator with pure oxygen until UVA_254_ was reduced by 25% (37 minutes of exposure) after which the effluent batches treated with ozone were mixed. Granular activated carbon (Hydraffin AR 8 × 30 diameter, 0.8–1 mm) was used as sorption material in the GAC treatment. The GAC treatment was done in two batches, with one of the tanks filled with 450 L of wastewater and 1850 g of activated carbon, the second tank was filled with 900 L of wastewater and 3700 g of activated carbon, reflecting an estimated GAC dosage of 4.1 g L^−1^. Again, the treatment with GAC was stopped when the UVA_254_ was reduced by 25%, (5 hours and 15 minutes of exposure). Directly after the treatment, the activated carbon particles were removed using a 0.425 mm filter, after which the effluent batches treated with GAC were homogenised by mixing. No additional (psycho)pharmaceuticals were added to the samples, and all analytes were already present in the effluent before treatment.

After treatment, 100 ml samples were collected in dark HDPE bottles (DS2185-0004, Thermo Scientific) using grab sampling. The samples from the Ozone treatment were taken from the untreated effluent before (“Pre-Ozone”, 2 samples) and after ozonation (“Post-Ozone”) from each batch (8 in total), while the samples from the GAC treatment were taken from the untreated effluent before (“Pre-GAC”, 2 samples) and after (“Post-GAC”) each batch (4 samples total), leading to 14 samples overall. All samples were stored at −20 °C and transported to the Institute for Biodiversity and Ecosystem Dynamics (IBED) at the University of Amsterdam (the Netherlands) for further analysis. Prior to analyses, samples were thawed overnight in 4 °C before extraction in October 2023.

### 2.3. Solid phase extraction

The extraction method used in this study is detailed in Davey *et al.*^25^ Briefly, after thawing, samples were spiked with labelled standards and shaken at 90 rpm for 30 minutes. Outlets, tubes, and adapters were cleaned with ultrapure water followed by methanol. Conditioning of the Oasis HLB cartridge (150 mg, 6 cc) was done with 6 mL MeOH and 6 mL ultrapure water. After sample loading (50 mL, in duplicate), the cartridge was dried for 30 minutes under vacuum and washed with 6 mL of ultrapure water. Before elution, a 0.22 μm polypropylene syringe filter was placed in between the cartridge and SPE inlet. Elution was achieved with 2 × 5 mL methanol under vacuum. The collected elution fractions were evaporated under a gentle nitrogen flow at 37 °C to <1 mL and reconstituted to 1 mL in methanol for storage until analysis.

### 2.4. UHPLC-HRMS method

The analytical method used here was the same as Davey *et al.*^25^ Briefly, a LC system (Nexera 30 Shimadzu, Den Bosch, The Netherlands) was coupled to a maXis 4G quadrupole time-of-flight HRMS (qToF/HRMS) upgraded with HD collision cell and ESI source (Bruker Daltonics, Leiderdorp, The Netherlands). The LC column used was an Acquity UPLC CSH C18 column (130 Å, 2.1 × 150 mm, 1.7 μm, Waters Corporation, Milford) kept at a temperature of 40 °C. The mobile phases consisted of ultrapure water (Milli-Q) with 0.05% acetic acid (A) and MeOH (B). The gradient started with a 7-minute equilibration at 10% B and gradually increased to 100% B in 10 minutes, held at 100% B for 5 min, and back to 10% B in 0.5 minutes, totalling 22.5 minutes. The flow rate was 0.3 mL min^−1^ and the injection volume 20 μL. For compounds above the linear quantification range, a second round of injections was performed with an injection volume of 5 μL. The samples were analysed in both positive and negative mode, acquiring HRMS1 spectra for 20–1000 *m*/*z* with a resolving power of 30 000–60 000 at full width half maximum (FWHM), with a spray voltage of +3.5 kV and −3.5 kV for positive and negative modes respectively.

Qualification and quantification of psychopharmaceuticals was carried out with TASQ version 2021.0 316 (Bruker Daltonics, Leiderdorp, the Netherlands). Qualification of target compounds was based on the mass accuracy of full-scan HRMS spectra and MS/MS ions acquired in data-independent MS/MS mode (DIA), and their retention time match with the calibration series.

During quantification, only chromatograms with a retention time (RT) of >1 minute, RT tolerance of ±0.3 minutes, mass tolerance of 0.002 Da, detectable qualifier ion, mSigma of <100, and a peak intensity of >1000, were considered (see SI-1 for further details). Calibration curves for quantification were calculated by analysing ultrapure water spiked with target compounds and serially diluted to obtain 18 concentration levels. The 18 solutions were further spiked to contain 10 μg L^−1^ of Internal Standard (IS). Concentration calibration was achieved using the calibration series in compared to calibration IS area to determine the per-sample recoveries and adjust for losses. For more information on the quantification method, see Davey *et al.*^25^ The instrumental and methodological limits of detection and quantification (LoD/LoQ) can be found in SI-5, columns Z-AA.

### 2.5. Method validation

The method for 30 selected psychopharmaceuticals was validated in a previous study^25^ while the method for an additional 5 pharmaceuticals was validated in the present study (SI-2). The method validation followed the same workflow as Davey *et al.*,^25^ where 5 sample types were made in order estimate the matrix effects and recovery efficiency for each of the 5 new pharmaceuticals. Only recoveries and matrix effects of between 60–140% were considered acceptable. The method validation results are reported in SI-2. Two compounds (caffeine and clonidine) were removed from further study due to contamination issues for caffeine and bad calibration results for clonidine (SI-3), thus the study included 33 compounds.

### 2.6. Removal efficiencies and preliminary risk analysis

After quantification, blank subtractions, and dilution adjustments, concentration-based removal efficiencies per compound were calculated using eqn (1) for both advanced treatment methods (SI-4):1
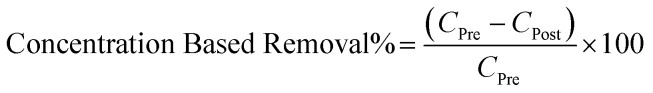
where *C*_Pre_ is the pre-treatment concentration, *C*_Post_ is the post-treatment concentration, and Concentration Based Removal% is the concentration-based removal expressed as a percentage.

A second removal calculation was also performed using the sum of the concentrations of all compounds per sample to allow direct comparison to risk-based removal (eqn (4)):2
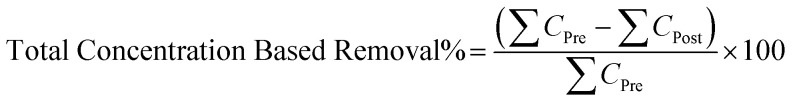
A preliminary risk analysis was performed to better understand the impact of the removal efficiencies by factoring the ecotoxicity of each compound to come to risk-based removal efficiencies. Risk quotients (RQs) were calculated per compound using eqn (3), based on the lowest available freshwater Predicted No Effect Concentration (PNEC) values taken from the NORMAN database (NORMAN, 2021).3
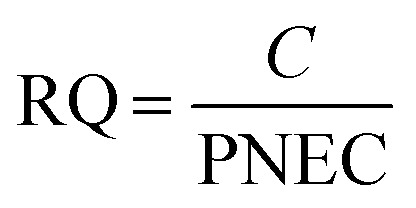
where *C* is the concentration (both pre and post treatment), PNEC is the predicted no effect concentration and RQ is the Risk Quotient.

Risk-based removal efficiencies for the treatments were then calculated in the same manner as total concentration-based removal efficiencies; however, using RQ instead of concentration (eqn (4), SI-5):4
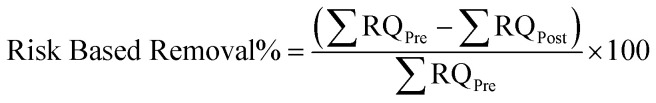
where RQ_Pre_ is the pre-treatment Risk Quotient, RQ_Post_ is the post-treatment Risk Quotient, and Risk Based Removal% is the risk-based removal expressed as a percentage.

Risk-based removal can only be calculated for the sum of the RQs, since the risk-based removal percentage would be identical to the concentration-based removal if done on a compound by-compound basis. Around 66% of (psycho)pharmaceuticals demonstrate additive mixture effects,^22,85^ thus the method of summing RQs for (psycho)pharmaceuticals has been argued for to bridge knowledge gaps.^86^

While the PNECs sometimes contained assessment factors according to the source used (SI-5, column C) no further assessment factors or dilution factors were applied during the preliminary risk assessment, contrary to what is done for deriving environmental quality standards.^87^ For compounds below limit of quantification (LOQ), or the limit of detection (LOD), the LOQ or LOD were used respectively for a worst-case risk calculation, which is then explicitly indicated in the results section.

### 2.7. Oxidation transformation product risk analysis

A literature search for expected oxidation transformation products (OTPs) from the ozone treatment was conducted for all parent compounds that returned a measurable concentration in the pre-treated effluent. The search was carried out in June 2024 using both Google Scholar and ScienceDirect using the search terms “<Compound Name>, Oxidation Transformation Product, OTP, Ozone, Pharmaceutical”.^58,60,63,66,88–101^

The OECD Toolbox was used to generate ecotoxicity data for the OTPs found in literature (QSAR Toolbox Version 4.7).^102^ Simplified Molecular-Input Line-Entry System (SMILES) or the chemical structure were used as input data for the QSAR toolbox (the SMILES for all OTPs are provided in SI-6). The lowest chronic ecotoxicity value (ChV) was taken from the QSARs for use as a PNEC. If the QSARs returned no ChV, or if the QSAR was outside of its applicability domain, then only an Effect Concentration (EC_50_) or a Lethal Concentration (LC_50_) was used with an assessment factor of 1000 to bring the EC_50_ or LC_50_ in line with the chronic ecotoxicity values. The QSAR PNECs were converted into molar PNECs to account for OTP mass differences, and the lowest OTP molar PNEC for each pharmaceutical (*i.e.* the most toxic OTP) was compared to molar PNEC of the parent to produce the toxicity difference between the parent compound and its most toxic TP (SI-6, column N). Using this relative worst-case toxicity factor of the OTP, a further risk assessment was performed to calculate the maximum risk possible if all removed parent compound would be transformed into the most toxic OTP as a worst-case scenario:5
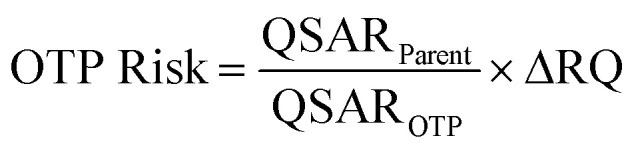
where: QSAR_parent_ is the molar PNEC as predicted by the QSAR model for the parent, QSAR_OTP_ is the molar PNEC as predicted by the QSAR model for the OTP, and ΔRQ is the change in RQ from the ozone treatment.

### 2.8. Statistical analysis

Paired T-tests were performed comparing the concentrations before and after treatment for each treatment type to test if significant removal had occurred. Since the ozone and GAC treatment occurred on separate days (see 2.2), a Mann–Whitney *U* test was performed as an independent non-parametric test comparing the removals of each treatment type. Analysis was performed in Microsoft Excel (see SI-7). Pearson correlations were run comparing the removal percentages of both treatments against structural molecular descriptors obtained from Chemicalize.^103^

## 3. Results and discussion

### 3.1. Concentrations and concentration-based removal efficiencies

Out of 33 target compounds 22 were detected in at least one sample, however clozapine and quetiapine returned unreliable results (SI-4), leaving the final of total quantified compounds as 20 out of 33. Concentrations range from 1 ng L^−1^ to 2.5 μg L^−1^ (SI-4). Hydrochlorothiazide, gabapentin, and pregabalin were the compounds with the highest concentrations, returning concentrations above 1 μg L^−1^ before treatment and remaining the highest concentrations after treatment. The effluent concentrations found in the present study are higher than in another recent Dutch study at a WWTP in Amsterdam.^25^ For example, carbamazepine is 5 times higher in the present study, and pregabalin and lamotrigine are one order of magnitude higher. The results in Bennekom, however, are in line with European medians.^25^

**Fig. 1:**
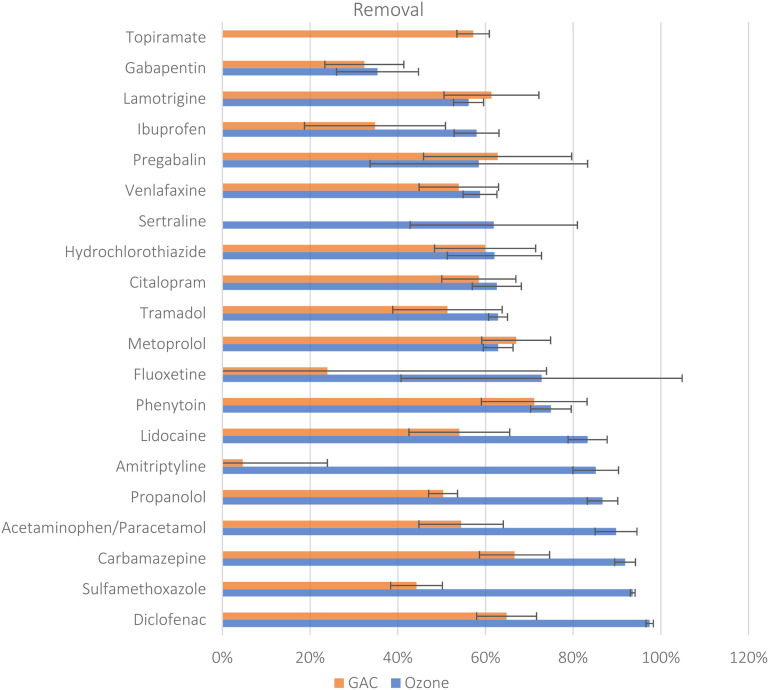
Median concentration-based removal efficiencies for the 20 quantified compounds calculated for treatment by GAC (*n* = 4) or ozone (*n* = 8), with error bars presenting the standard deviation. Negative removals for topiramate (ozone = −128%) and sertraline (GAC = −7%) are not shown in this figure.

Fig. 1 shows the concentration-based removal efficiencies for the 20 quantified compounds, where removals range from −128% to 97% (median 63 ± 5%, *p* < 0.05) for ozone, and from −15% to 71% (median 54 ± 9%, *p* < 0.05) for GAC. The Mann–Whitney *U* test showed a significant difference between the removals of the two treatment types (*p* < 0.05, *r* = 0.37, SI-07*).* Two compounds returned a higher median concentration after treatment and thus showed negative removal; these were topiramate with −128 ± 57% in ozone and sertraline with −15 ± 8% in GAC. For these compounds deconjugation cannot be an explanation for the negative removal, since neither of these compounds have conjugated metabolites.^28,104^ Analytical artefacts due to the proximity to the LOQ of these two compounds may explain the negative removal. Large standard deviations in removals (Fig. 1) of fluoxetine and amitriptyline, for example, are due to one or more of the samples being <LOQ, see SI-4 for full results.

The Pearson correlations indicated that for ozone, the number of activated aromatic rings correlated with better removal (*r* = 0.65), while the fraction of carbon atoms that are sp^3^-hybridised negatively correlated with removal the most (*r* = −0.63). This confirms that ozone is behaving as expected in this study, as the electrophilic ozone can attack the electron rich activated aromatic rings (*e.g.* diclofenac, carbamazepine, paracetamol, *etc.*, Fig. 1), while it struggles to attack sp^3^ bonds, with compounds that lack aromatic rings and are sp^3^ rich, such as gabapentin and pregabalin, showing the lowest removal efficiencies by ozone (SI-8). The correlations agree with the qualitative analysis by Zoumpouli *et al.*^79^ (2020), which identified reactive and non-reactive functional groups in ozone removal. For GAC, topological polar surface area correlated strongest with removal (*r* = 0.54), while log *K*_ow_ correlated the most negatively (*r* = −0.46). Again, this is in line with what can be expected for an absorptive material, with compounds with higher polar surface areas able to interact with the surface of the GAC, while compounds such as amitriptyline are non-polar and very lipophilic returning very low GAC removals (Fig. 1). Since ozone favours electron-rich aromatics and GAC favours polar molecules, the two treatment types can target different compounds, as shown by the inverse of the Pearson correlation between the two treatment types across all the molecular descriptors (SI-8).

Making direct comparisons to removal efficiencies found in literature remains difficult, due a lack of standardisation in testing methodologies^45,78^ and differences in dosages between studies.^45–48,50,65,70,72,76,77,79–82^ The dosage and exposure time used this study have been shown to produce removals of >80% for micropollutants in studies with dosages and exposure times.^47,82,83^ The results presented here for removal by ozonation are broadly in line with other literature. Similar removal rates to Bourgin *et al.*^44^ after ozone treatment were found for carbamazepine, diclofenac and sulfamethoxazole (>90% removal, SI-4), and compared to Spilsbury *et al.*^70^ the present study had significantly better ozone removal at a comparable dose of 0.35 g O_3_ g^−1^ DOC. However, for other compounds, removal efficiencies were slightly lower than those of other lab-, or pilot-scale experiments.^27,44,105^ For GAC, removal efficiencies found here were substantially lower than those reported in literature. This is likely due to the lower dose used in the present study compared to literature,^27,77^ as well as the lack of a biofilm in the present study. GAC is commonly implemented as a fixed bed adsorber with biofilm development that can provide additional biodegradation over longer operation.^77^ Here, virgin GAC was used as a purely adsorptive substrate, with the dosage and contact time being similar to other GAC studies.^50,81^ However, using Powdered Activated Carbon (PAC) to increase sorption surface area, or extending the operation of GAC to allow for a biofilm to form, incurring biodegradation potential may improve the removals presented here.^48,49,77^

### 3.2. Risk analysis

**Fig. 2:**
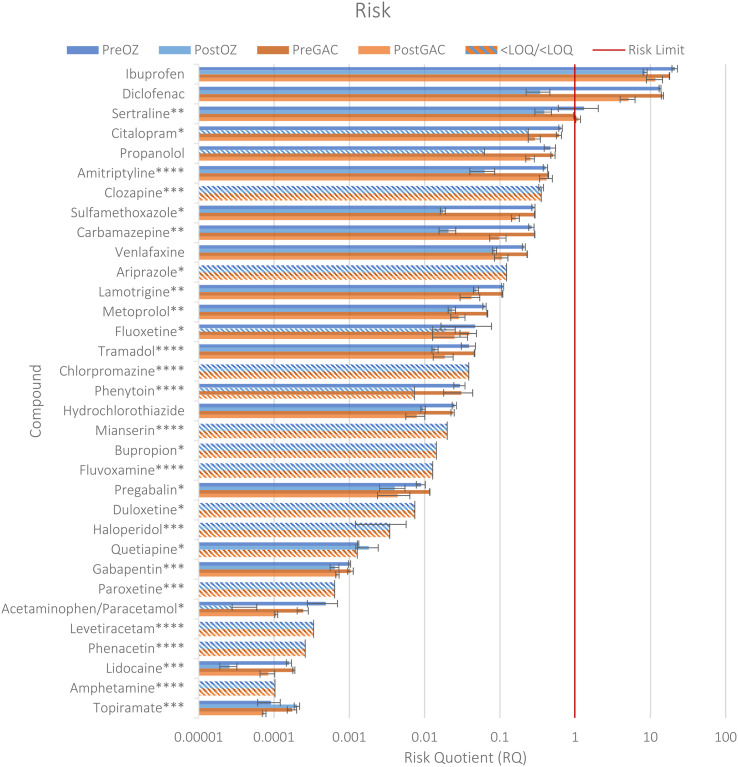
Risk quotients for all 33 compounds in the study, for all 4 samples. The red line indicates where a risk is seen (*i.e.* RQ > 1) and the error bars indicate the standard deviation. Striped bars indicate that the risk assessment was based on the LOD or LOQ. Asterisks next to the names indicate the assessment factor used by the NORMAN database. * = 10, ** = 50, *** = 100, **** = 1000 (QSARS only), no asterisk indicates no assessment factor present.

Ecotoxicological preliminary risks for the parent compounds were calculated for all 33 compounds present in the study (Fig. 2, SI-5), since LOD and LOQ values were used as surrogates for the compounds that could not be quantified or detected, to present a worst-case scenario. Here, ibuprofen, diclofenac, and sertraline present a risk in the untreated effluent (RQs of 21, 13, and 1.3 respectively). Diclofenac, citalopram, amitriptyline, clozapine, carbamazepine, venlafaxine, sulfamethoxazole, aripiprazole, propranolol, and lamotrigine had RQs of over 0.1, meaning they were within 1 order of magnitude to a risk. Aripiprazole was within an order of magnitude to an RQ of 1 despite all measurements being below the LOQ, due to the small distance between the potentially toxic concentrations and the LOQ. The median RQs were 0.025, 0.013, 0.023, 0.014 for Pre-Ozone, Post-Ozone, Pre-GAC, and Post-GAC respectively.

The PNECs obtained from the NORMAN database originate from diverse sources. While many were from literature, 9 out of 30 were QSAR predicted, and the assessment factors used in the NORMAN database ranged from 0 to 1000 (SI-5). The use of the NORMAN PNECs was a pragmatic choice due to their availability, however the shortcomings of these PNECs has been discussed before.^25^ The risk assessment performed here is illustrative and not designed to be compared to EMA style risk assessments, since the ecotoxicity data for those are generally lacking.^23,24,69^ For the mentioned highest risk compounds, the PNECs were based on literature data, and had assessment factors of only 10–50 applied (Fig. 2).^106^

### 3.3. OTP toxicity and QSAR data gap filling

**Fig. 3:**
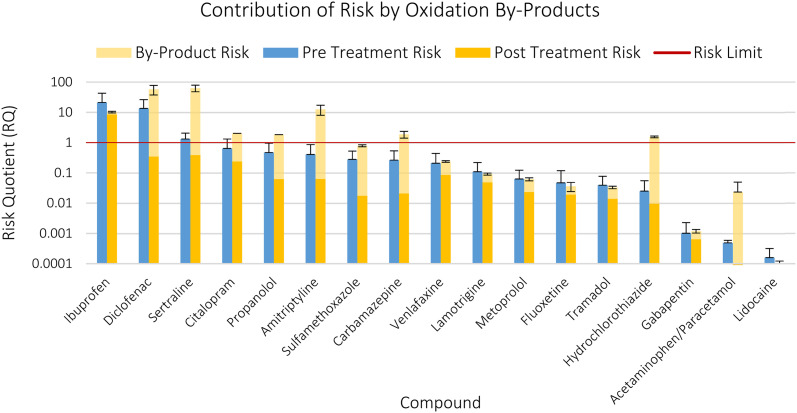
Risk quotients of 17 detected parent compounds including a worst-case risk estimation of probable OTPs after ozonation. Error bars indicate standard deviation.

146 structures of known OTPs that might be formed by ozone treatment were obtained from literature for 17 of the 22 detected compounds (SI-6).^58,60,63,66,88–101^ The number of OTPs per parent compound range from 4 to 30. Most of the OTPs returned ChVs, with only 18 OTPs returning an EC/LC50 value and requiring an assessment factor (SI-6). Based upon the QSAR calculations, the risk of most OTPs that could be produced by the ozone treatment is greater than the risk that was removed by it, where a total of 29 OTP structures returned ecotoxicity values lower than their parents, and thus more toxic. For diclofenac, sertraline, citalopram, propranolol, amitriptyline, sulfamethoxazole, carbamazepine, venlafaxine, hydrochlorothiazide, gabapentin, and paracetamol the possible contribution of the most toxic OTP to the toxicity of the effluent was higher than the toxicity by the parent compound that was removed (Fig. 3, SI-6). In the case of paracetamol, the most toxic OTP was around 100 times more toxic than the parent compound on molar basis. As a result of the OTP analysis, citalopram, amitriptyline, carbamazepine, and hydrochlorothiazide went from no predicted risk based only on the parent compound, to a predicted risk based on parent and OTP after ozonation (Fig. 3).

The formation of ecotoxic OTPs has been discussed in literature^58,60,62^ but the exact effects of individual OTPs in these studies are difficult to unravel due to the lack of OTP standards, and most studies on OTP toxicity being broad-scope bioassays on ozonated water.^62^ The analysis performed here attempted to bridge this gap by estimating the individual OTP toxicity using *in silico* methods and is broadly in agreement with literature that the formation of OTPs can be detrimental to the ecosystem without an additional treatment step to remove them. However, it should be reiterated that the actual concentrations of the OTPs were unknown, and thus the analysis performed and shown in Fig. 3 should be seen as a worst-case scenario which may not reflect the real situation.

When assessing the risk of pharmaceuticals, many studies neglect transformation products for practical reasons.^21,25^ The current lack of analytical standards leads to large uncertainties in quantification, and including analytes without standards is often advised against.^107^ Additionally, the lack of standards leads to a lack of ecotoxicity data for these transformation products. While the use of QSARs on the confirmed OTP structures provided data to fill gaps, there is still many missing data to accurately estimate the risk after ozonation. Firstly, there are probably more OTP structures than those reported in literature,^59^ and there were some compounds with missing literature on OTP structures (SI-6). There are also the OTP structures of human metabolites of the psychopharmaceuticals, which were available only for some pharmaceuticals,^91,92,95,97,101^ and which are not included here but could add to the risks. Secondly, there are some general limitations with the use of QSARs. Quality and performance of the models are highly dependent on the training dataset and thereby applicability domain.^108^ However, none of the QSARs used in the present study were outside the applicability domain. Toxicological mode of action is also a limitation for QSARs. Like the PNECs, behavioural endpoints are not covered in the QSARs, although they might be vital to understanding the ecological impact of psychopharmaceuticals.^16,109^

### 3.4. Risk-based removal efficiencies

Comparing total concentration-based removal efficiencies to risk-based removal efficiencies when not factoring in OTP toxicity, the summed median concentration-based removal efficiency across all compounds was 60 ± 3% for ozone yielding a 73 ± 2% risk-based removal, while the summed median concentration-based removal efficiency for GAC is 57 ± 9% and 46 ± 11% in terms of risk reduction. When factoring in the worst-case OTP toxicity, the risk-based removal efficiency for ozone flips to −274 ± 124%, indicating that there may be an increase in risk during ozonation. Thus, ozone is slightly better at removal of parent compounds in terms of mass, but due to the expected creation of OTPs the risk to the environment is potentially even increased after ozonation (SI-5).

The use of risk as a method of contextualising removal efficiencies is a novel approach^25,52,70^ as often toxicity is not factored into studies on removal efficiencies of treatment technologies. Additionally, policy goals are often expressed as removal efficiencies in terms of concentration and not in terms of risk.^44,49,73,74^ However, the worst-case risk analysis in this work reflects a scenario where risk is evaluated in terms of PNECs for standard ecotoxicity test endpoints such as growth, reproduction, or mortality, as well as the known limitations of the PNECs which have been discussed before.^25^ This includes the lack reflection of more complex interactions such as behavioural effects in available ecotoxicity studies, or ecosystem dynamics such as changes in food webs, for which effects can be non-monotonic.^16,17^ Availability of such data would increase the accuracy of the risk assessment but also add to the complexity of the assessment.^21,110^ Still, the risk-based evaluation of removal efficiencies by treatment technologies used in the current study has clear benefits over traditional concentration-based removal efficiencies, as it relates much better to the endpoint of a protected and healthy ecosystem. Such a risk-driven approach has been discussed as a model for policy makers but is not yet applied generally in policies.^73,111^

Despite the specified limitations, the present study demonstrates the value in using models to fill in for missing experimental ecotoxicity data, even if the results should be interpreted as more qualitative than quantitative. Ecotoxicity tests are costly, time consuming, and may provide a limited view on risks related to ecological functioning and have ethical questions attached to them. Modelling can negate some of these concerns and allow for far more compounds to be included in the analysis, elucidating potential risks. Combined with higher throughput laboratory methods, such as non-target screening tools with structure generation,^112^ and novel semi-quantitative LCMS methods,^113^ there is a potential for a high throughput risk assessment to be performed using new and existing LCMS occurrence data, or even modelled data.^114^

### 3.5. Implications for wastewater treatment

In this study, ozone outperformed GAC both in terms of concentration-based removal and in terms of risk-based removal (SI-5). However, the advantage of ozone may disappear due to the formation of toxic OTPs, which is also reflected in the literature.^58,60,62^ The inverse of the Pearson correlations between the two treatment types across the molecular descriptors indicates that GAC works well on compounds which ozone does not (SI-8), which has also been shown in other studies.^77^ This is why in practice ozone and other AOPs are in tandem with an adsorptive technique such as GAC, PAC, or other biofiltration methods.^60^ This type of tandem setup is already used in many existing and pilot advanced treatment setups for especially drinking water treatment and to a lesser extent wastewater treatment.^65,115–119^ However, there are still arguments against the deployment of widespread advanced treatments, citing the upfront and running costs of advanced treatments. This argument is compounded by the lack of tertiary treatments (*e.g.* CAS) in large parts of Europe,^27^ which should be the priority in those regions. Some estimations indicate that advanced treatment costs can be up to two times the current operating costs of a WWTP, with GAC being more expensive than ozone to implement.^43^

Lowering the operating costs of advanced treatments can lower the barrier to their adoption,^120^ which may be possible by lowering dosages if it can be assured that the risks are sufficiently low, even when taking temporal variation of influent concentrations into account.^121^ Utilising risk-based removal, or other risk-based approaches, as have been discussed in the EU,^73^ could provide a potential way to reduce the dose of advanced treatments. A potential workflow for reducing advanced treatment dosage could use the UVA_254_ method alongside non-target and semi-quantitative methods used in tandem with OTP structure and toxicity prediction perform a preliminary risk assessment on OTPs.^59,112,113,122^ If done on a tandem ozone-GAC setup, the dosage of both ozone and GAC, as measured by UVA_254_, could be raised or lowered based on predetermined risk thresholds of both parent compounds and OTP. This workflow could allow for a more dynamic system when compared to the current flat, percentage-based removal targets.^73^

However, major knowledge gaps would need to be addressed first, such as robust ecotoxicity data,^23,123^ and concerns over the identity, quantity and toxicity of biological metabolites and OTPs of the psychopharmaceuticals. It may be possible to also use bioassays and effect-based studies as combined with effect directed analysis to aid in answering the question of ecotoxicity of OTPs after ozonation^124–130^ with an alternative being broader mesocosm-style experiments.^17,131^

## 4. Conclusions

The present study investigated a risk-driven approach to directly assess the environmental risk and removal efficiencies of two advanced wastewater treatment technologies on (psycho)pharmaceuticals, *i.e.* ozone and GAC. Ozone performed better in terms of both concentration-based removal and risk-based removal for parent compounds. When QSARs were utilised to fill ecotoxicity data gaps on OTPs in a worst-case scenario, the results give a strong indication that ozone produces toxic byproducts that not only negate the risk reduction but may even increase the toxicity of the ozonated effluent. Furthermore, analysis on molecular descriptors indicates that ozone and GAC can remove different, complementary groups of (psycho)pharmaceuticals. Combined with the concerns over OTP toxicity, a tandem ozone-GAC setups would therefore be recommended. Finally, the study demonstrates the merits of novel methodologies, namely the UVA_254_ method for direct comparison of different treatment methods, and the risk-driven approach over flat concentration-based removal targets as a metric for removal. Future workflows should include semi-quantitative risk assessment for OTPs to further elucidate the potential risk of these transformation products.

## Conflicts of interest

There are no conflicts of interest to delcare.

## Supplementary Material

EW-011-D5EW00600G-s001

## Data Availability

Supplementary information is available. See DOI: https://doi.org/10.1039/D5EW00600G. The data supporting this article have been included as part of the supplementary information (SI).
